# Effect of enriched housing on levels of natural (auto-)antibodies in pigs co-infected with porcine reproductive and respiratory syndrome virus (PRRSV) and *Actinobacillus pleuropneumoniae*

**DOI:** 10.1186/s13567-017-0481-y

**Published:** 2017-11-10

**Authors:** Lu Luo, Ingrid Daniëlle Ellen van Dixhoorn, Inonge Reimert, Bas Kemp, Jantina Elizabeth Bolhuis, Hendrik Karel Parmentier

**Affiliations:** 1 Adaptation Physiology Group, Department of Animal Sciences, Wageningen University & Research, P.O. Box 338, 6700 AH Wageningen, The Netherlands; 2 Wageningen Livestock Research, P.O. Box 338, 6700 AH Wageningen, The Netherlands

## Abstract

Housing of pigs in barren, stimulus-poor housing conditions may influence their immune status, including antibody responses to (auto-)antigens, and thus affect immune protection, which will influence the onset and outcome of infection. In the present study, we investigated the effects of environmental enrichment versus barren housing on the level of natural (auto-)antibodies (NA(A)b) and their isotypes (IgM and IgG) binding keyhole limpet hemocyanin (KLH), myelin basic protein (MBP), and phosphorycholine conjugated to bovine serum albumin (PC-BSA) in pigs co-infected with porcine reproductive and respiratory syndrome virus (PRRSV) and *Actinobacillus pleuropneumoniae* (*A. pleuropneumoniae*). Pigs (*n* = 56) were housed in either barren or enriched pens from birth to 54 days of age. They were infected with PRRSV on 44 days of age, and with *A. pleuropneumoniae* 8 days later. Blood samples were taken on 7 different sampling days. Housing significantly affected the overall serum levels of NA(A)b binding KLH, MBP and PC-BSA, and before infection barren housed pigs had significantly higher levels of NA(A)b than enriched housed pigs, except for KLH-IgM and PC-BSA-IgG. Infection only affected the IgM, but not the IgG isotype. Moreover, changes in MBP-IgM and PC-BSA-IgM following infection were different for enriched and barren housed pigs. These results suggest that the effect of infection on NA(A)b is influenced by housing conditions and that NA(A)b, especially IgM may be affected by infection.

## Introduction

Commercial pigs usually live in barren, stimulus-poor housing conditions. The limited possibilities to express important behaviours under such conditions may lead to signs of chronic stress [1–3], and the development of damaging behaviours directed at group members, such as tail or ear biting [4–6]. It has been suggested that housing, stress and social factors are associated with disease susceptibility and therefore environmental conditions have been indicated as cofactors in the pathogenesis of infectious diseases [7, 8]. Several studies reported immunological differences between pigs kept in barren conditions as compared to pigs provided with environmental enrichment, such as substrates to manipulate, or extra space [9–11]. Moreover, a recent study [8] reported that pigs kept in enriched pens, showed a faster viral clearance and were less likely to develop lung lesions following a porcine reproductive and respiratory syndrome virus (PRRSV) and *Actinobacillus pleuropneumoniae* (*A. pleuropneumoniae*) co-infection as compared to barren housed pigs. PRRSV and *A*. *pleuropneumoniae* are pathogens that are frequently involved in the porcine respiratory disease complex (PRDC), which causes severe health and welfare problems, and major economic losses in swine industry [12].

We recently demonstrated that enriched housed pigs had higher levels of natural autoantibodies binding danger (phosphorycholine, PC) and neural (myelin basic protein, MBP) auto-antigens than barren housed pigs [13]. PC is a component of cell membranes and levels of anti-PC antibodies may reflect cell damage and inflammation related to stress [14]. MBP is an important protein in the nervous system, and levels of antibodies binding MBP were affected by mental stress [15]. Furthermore, Reimert et al. [16] reported that enriched housed pigs had higher levels of natural antibodies (NAb) binding keyhole limpet hemocyanin (KLH) than barren housed pigs. KLH is an antigen without known cross reactivity with mammalian antigens and it has been widely used to detect natural antibodies in a variety of species, including pigs [16]. NAb appear in the absence of apparent antigenic stimulation and are secreted by long lived, self-renewing B1 cells. NAb have been proposed to be an essential part of the first line of defence against viral and bacterial infections [17–19]. A large part of NAb (70–80%) binds to auto-antigens. Due to low affinity and poly-specificity, both natural IgM and natural IgG may protect the host by binding to bacteria and viruses by enhancing phagocytosis. IgM antibodies play a role in clearing apoptotic cells, maintain B cell homeostasis [20], and can simultaneously bind to different conserved structures, like PC, nucleic acids, and carbohydrate on the same pathogen and inhibit the pathogen from invading cells and dispersing into different organs [19, 21]. Natural IgM may thus limit the spread of infection [22, 23], and as such provide defence against viral infections directly by inhibiting spread of virus or by T cell activation or by binding with viral and chemokine receptors [22]. IgG can recognize a range of Gram-negative and Gram-positive bacteria with the aid of serum lectin pattern recognition receptors (PRRs) which are known to bind to sugar residues on the bacteria. In this way, IgG can collaborate with lectin, thus swiftly and effectively kill invading pathogens [24]. It has been proposed that natural IgM-producing B cells do not undergo affinity maturation or activation, and subsequently shift to IgG, but in contrast to antigen-specific immunoglobulin producing B2 cells, provide a readily available poly-specific source of defence, in response to activation of innate receptors, like TLRs [19].

Studies reported that levels of NA(A)b may rise with age but are independent of the (antigenic) environment. However, in a former study, effects of housing conditions on levels of NA(A)b were found [13]. In the current study we studied the effect of housing and a co-infection with PRRSV and *A. pleuropneumoniae* on levels of natural IgM and IgG binding KLH, MBP and PC-BSA. To that aim, we studied first, before infection, the effects of housing on these NA(A)b and their changes in time, and second, after infection, the interactive effect of infection and housing on NA(A)b changes.

## Materials and methods

The established principles of laboratory animal use and the EU and Dutch laws related to animal experiments were adhered to in this study.

### Animals and housing

We studied serum samples from 56 male and female piglets (Topigs 20 line, from Great Yorkshire × Landrace origin), the same pigs as described in [8]. In short, piglets were offspring of 8 multiparous sows obtained from a PRRSV and *A. pleuropneumoniae*-free herd. Sows were inseminated on the same day and the expected parturition day was defined as day 0 for all piglets. Sows were housed in farrowing crates at research facility A of Wageningen University Livestock Research, the Netherlands from 2 weeks before parturition. From the first day of life onwards, half of the pigs were housed in conventional 5 m^2^ barren pens with 100% slatted floor and a 100 × 45 cm solid rubber floor mat. The other half of the pigs were housed in 10 m^2^ enriched pens with partly slatted (40%) and partly solid (60%) floors. In both the enriched and barren pens, two chains with blocks were added as enrichment. Two jute bags and branches of a broom were provided in the enriched pens, and they were replaced weekly. Additionally, 1 kg straw, 160 L of moist peat and 180 L of wood shavings were provided in the enriched pens as rooting substrate. Straw and wood shavings were replenished daily (0.5 kg/day straw, 23 L/day of wood shavings), and 20 L of fresh peat was added weekly. From 13 days of age until weaning, the panels between two adjacent enriched pens were removed. Thus, the four individual enriched pens of 10 m^2^ were temporarily transformed into two pens of 20 m^2^ to enable early social interaction between two enriched-housed litters. Each pen was cleaned daily and enrichment materials and food were γ-irradiated (9 kGy irradiation; Synergy Health Ede BV, the Netherlands). During the first week after birth, a heating lamp was provided in each pen. Each pen had two drinking nozzles, one for the sow and one for the piglets. Sows were fed a standard commercial diet twice a day. From 3 days of age, the piglets received solid food ad libitum. Lights in the pens were on between 6:00 a.m. and 6:00 p.m., and the temperature was 25 °C, and gradually decreased to 22 °C the week before weaning.

### Weaning, regrouping and relocation

Piglets were selected at weaning (31 days of age). Seven piglets per litter were selected to regroup into 8 new experimental groups. All piglets were equally mixed, meaning an equal number of unfamiliar pigs per group and new groups with comparable compositions were formed. Gender, bodyweight, and coping style (see [8]) were taken into account in the selection of piglets, and housing treatment (barren vs. enriched) for each piglet was kept the same as before weaning.

At 39 days of age, all piglets were transported to facility B of Wageningen University Livestock Research, the Netherlands. Pigs from 2 barren and 2 enriched groups were housed in separate High Efficiency Particulate Air (HEPA) filtered animal rooms, and the other 2 barren and 2 enriched groups were kept in separate pens within one large room without extra biosafety measures as negative-controls. Housing conditions, group structure, pen sizes and access to food and water remained the same as before transport.

On the day of weaning, the temperature was 28 °C and was decreased by 2 °C each week until it reached 22 °C. It was kept at 22 °C until the end of the experiment.

### Infection procedure

At 44 days of age, half of barren and enriched housed pigs were inoculated intra-nasally with 1.5 mL inoculum containing 5 log_10_ 50% tissue culture infectious dose (TCID_50_) of the mild-virulent European PRRSV serotype I strain LV-Ter Huurne. At 52 days of age, pigs were infected with *A. pleuropneumoniae* by aerosol. See for more details [8].

Half of the barren control and enriched control pigs were not inoculated (negative controls) and underwent no extra handlings. The other half of barren control and enriched control pigs underwent the same procedures as the infected pigs (mock control) at the same days. Instead of PRRSV inoculum and *A. pleuropneumoniae*, mock control pigs received 1.5 mL RPMI medium and 5 mL PBS, respectively.

### Blood collection and enzyme-linked immunosorbent assay

Blood samples were taken by jugular vein puncture on 7 different days (38, 44, 46, 48, 52, 53, and 54 days of age). Blood was collected in serum separating tubes (Greiner Bio-one, Alphen aan den Rijn, The Netherlands) kept at room temperature (RT) until incubation for 1 h at 37 °C. After that samples were centrifuged at 5251 *g* for 12 min at −20 °C. Serum was stored at −80 °C until analysis.

IgM and IgG antibody titers binding with keyhole limpet hemocyanin (KLH), myelin basic protein (MBP, Sigma-Aldrich, St. Louis, MO, USA) or Phosphorylcholine-conjugated to Bovine Serum Albumin (PC-BSA, Santa Cruz Biotechnology, Santa Cruz, CA, USA) were determined by a two-step indirect enzyme-linked immunosorbent assay (ELISA). Medium binding microtiter plates (Greiner) were coated overnight at 4 °C with 2 mg/mL KLH or 0.5 µg/mL PC-BSA or 0.5 µg/mL MBP in coating buffer (0.05 M Na_2_CO_3_ × 10 H_2_O, pH 9.6). Plates were washed with tap water containing 0.05% Tween 20, and serial dilutions of serum were added and incubated for 1.5 h at RT. After washing, plates were incubated for 1.5 h at RT with a 1:40 000 diluted PO-conjugated goat antibody directed to swine IgM_FC_ (GASwIgM_FC_/PO, Bethyl Laboratories, Montgomery, TX, USA) to detect binding of IgM, or with a 1:40 000 diluted peroxidase (PO)-conjugated goat antibody directed to swine IgG_FC_ (GASwIgG_FC_/PO, Bethyl Laboratories) to detect binding of IgG, respectively. After washing, tetramethylbenzidine was added as a substrate for 10 min. Reaction was stopped with 2.5 N H_2_SO_4_ and absorbance was measured at 450 nm with a Multiskan (Flow, Irvine, UK). Each absorbance was expressed relatively to the absorbance of a standard positive control serum sample, and antibody titers were expressed as log_2_ values of dilutions that gave extinction closest to 50% of E_max_, where E_max_ represents the highest mean extinction of a standard positive serum present on every microtiter plate.

### Statistical analyses

SAS (SAS 9.3, SAS Institute Inc.) was used for all statistical analyses. In this experiment, the mock-infected control groups were used to make sure that the procedure to infect the pigs alone did not cause any differences. Preliminary statistical analysis (mixed model) showed no differences in outcome between mock-infected and negative control pigs, so these groups were combined and referred to as “control group”.

#### Overall antibody response

Antibody titers were analysed using a repeated linear mixed model. The fixed effects of housing, infection, sampling day, and their interactions were included in the model, as well as the random effect of animal. Values in time of individual animals were taken as repeated measurements. All data met the assumptions of the model regarding normal distribution of residuals. Subsequently, separate analyses were performed on the housing effect before infection, the housing effect on changes in antibody titers after transport and relocation and the effects of infection and its interaction with housing on changes in antibody titers after infection (see below).

#### Housing effect on antibody titers before infection

Values on day 38 were used to analyse the effects of housing before transport and infection with a linear mixed model with the fixed effect of housing.

#### Housing effect on changes in antibody titers after transport and relocation

To investigate housing effects after acute stress due to transport and relocation, the delta between day 44 and day 38 was calculated and subsequently analysed with a linear mixed model with the fixed effect of housing.

#### Effects of infection and its interaction with housing on changes in antibody titers after infection

To investigate effects of infection, the deltas in titers between day 44 (before PRRSV infection) and days 46, 48, and 52 were calculated for the infection of PRRSV, and the deltas between day 52 (before *A*. *pleuropneumoniae*, infection) and days 53 and 54 were calculated for the infection of *A*. *pleuropneumoniae*, respectively, and separately analysed with a linear mixed model with housing, infection, delta-day and their interaction as fixed effects, and a random effect of animal. Values in time of individual animals were taken as repeated measurements.

To explore relationships between NA(A)b responses and PRRSV infection characteristics, correlations between basal NA(A)b levels on day 44 (start of infection) and the NA(A)b increase from day 44 to 52 (delta 44–52) with viral RNA measured on 52 of infection, and the decrease in viral RNA from day 48 to 52 were assessed. Data on viral RNA in the same pigs were available from and have been reported in a previous study [8]. In case of significant correlations, differences between correlation coefficients of barren and enriched housed pigs were calculated using Fisher’s *r*-to-*z* transformation. Variables of viral RNA could not be obtained from all 28 infected pigs because of technical problems [8].

Only significant effects (*P* < 0.05) and tendencies (*P* < 0.10) are reported. Significant interactions were further investigated with post hoc pairwise comparisons using the difference of the least square means with Tukey adjustments. Results are presented as mean ± SEM.

## Results

### Natural (auto)-antibody titers

**Figure 1:**
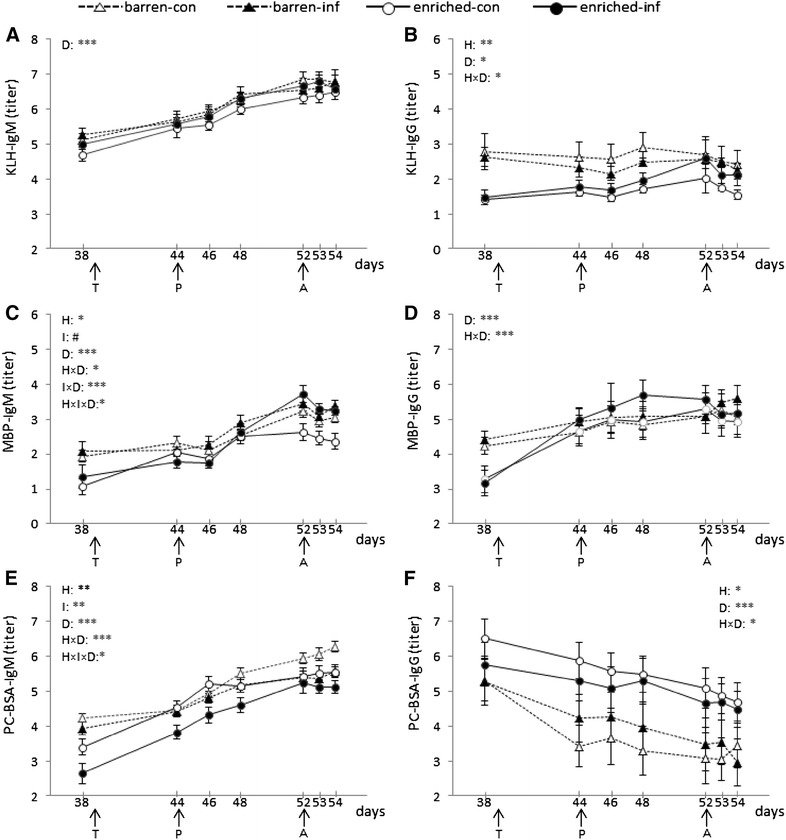
**Means and SEM of antibody isotype titers.** IgM (**A**) and IgG (**B**) binding KLH, IgM (**C**) and IgG (**D**) binding MBP, and IgM (**E**) and IgG (**F**) binding PC-BSA of pigs (*n* = 56) in barren and enriched housing conditions measured on 7 different days. At 39 days of age, pigs were transported to the experimental rooms (T); at 44 days of age, BI and EI pigs were infected with PRRSV (P); at 52 days of age, BI and EI pigs were infected with A. *pleuropneumoniae* (A). H indicates the housing effect; I indicates the infection effect; D indicates the sampling day effect. ***P < 0.001, **P < 0.01, *P < 0.05, ^#^ *P* < 0.10.

**Figure 2:**
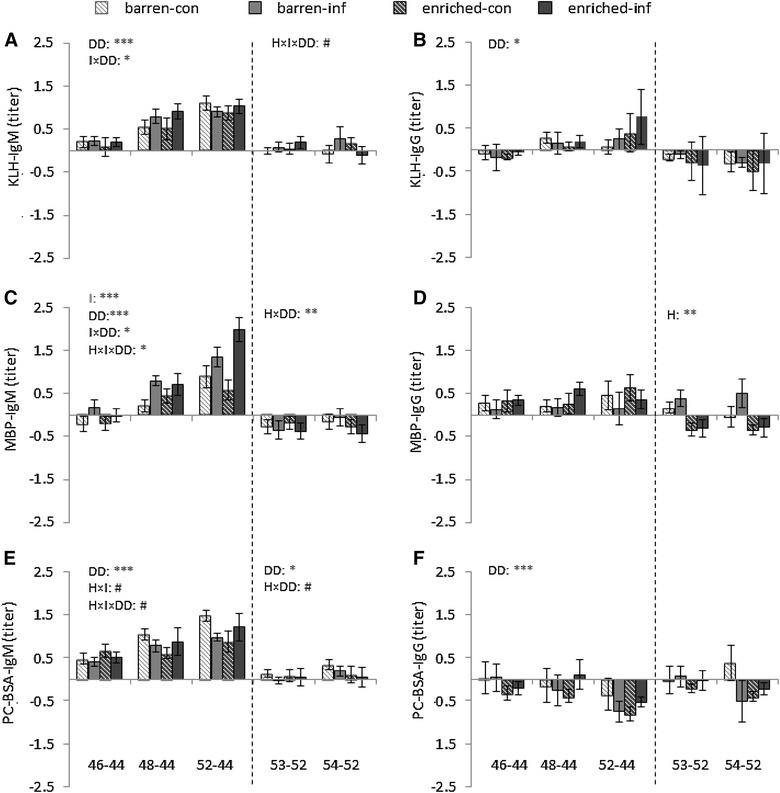
**Means and SEM of the delta in antibody titers between infection days and sampling days.** IgM (**A**) and IgG (**B**) binding KLH, IgM (**C**) and IgG (**D**) binding MBP, and IgM (**E**) and IgG (**F**) binding PC-BSA of pigs (*n* = 56) in barren and enriched housing conditions for delta (46–44, 48–44, 52–44, with day 44 = PSSRV infection) and delta (53–52, 54–52, with day 52 = A. *pleuropneumoniae* infection). The dashed lines divide each graph into the two separate infection conditions. H indicates the housing effect; I indicates the infection effect; DD indicates the delta-day effect. ***P < 0.001, **P < 0.01, *P < 0.05, ^#^ *P* < 0.10.

The course of the antibody titers from day 38 until day 54 is shown in Figure 1. Apart from this, the responses to transport and relocation (delta day 44–day 38) are shown in Table 1. Finally the responses to the PRRSV infection (delta from day 44 = start of infection) and to *A. pleuropneumoniae* (delta from day 52 = start of A. pleuropneumoniae infection) are shown in Figure 2.

**Table 1:** **Means and SEM of changes (delta 44–38) in antibody isotype titers of barren and enriched housed pigs before and after transport/relocation**

	Barren	Enriched	*p* value
KLH-IgM	0.50 ± 0.11	0.76 ± 0.18	ns
KLH-IgG	−0.32 ± 0.19	0.35 ± 0.11	*
MBP-IgM	0.26 ± 0.13	0.70 ± 0.15	*
MBP-IgG	0.46 ± 0.21	1.58 ± 0.31	**
PC-BSA-IgM	0.48 ± 0.13	1.37 ± 0.24	**
PC-BSA-IgG	−1.45 ± 0.22	−0.67 ± 0.18	**

Effects of housing after transport/relocation (delta 44–38) are reported in the text; significances of housing are indicated: ** *P* < 0.01, * *P* < 0.05, and ns for non-significant.

### IgM and IgG binding KLH

#### KLH-IgM

*Overall antibody response* Overall, KLH-IgM titers were not affected by housing or infection, but only by sampling day (F(6.307) = 90.8, *P* < 0.001), with increasing titers from day 38 to 52 (only day 44 and day 46 titers did not differ), but after infection with *A. pleuropneumoniae* on day 52, levels of KLH-IgM remained stable (Figure 1A).

*Housing effect on antibody titers before infection* On day 38 (before transport), housing tended to affect KLH-IgM (F(1.54) = 3.5, *P* < 0.10), with higher titers in barren than in enriched housed pigs (Figure 1A).

*Housing effect on changes in antibody titers after transport and relocation* There was no housing effect on the changes in KLH-IgM before and after transport (Table 1).

*Effects of infection and its interaction with housing on changes in antibody titers after infection* After infection with PRRSV on day 44, changes of KLH-IgM (delta 46–44, 48–44, and 52–44) were not only affected by delta-day (F(2.99) = 75.6, *P* < 0.001), but also by the interaction between infection and delta-day (F(2.99) = 3.4, *P* < 0.05). Post hoc analysis showed that the changes of titers in control pigs significantly increased from 46–44 to 48–44 to 52–44, whereas in infected pigs, changes of titers increased from day 46–44 to 48–44, with no difference between 48–44 and 52–44 (Figure 2A). Besides, changes in KLH-IgM after infection with *A*. *pleuropneumoniae* tended to be affected by the housing × infection × sampling day interaction (F(1.50) = 3.1, *P* < 0.10), with no clear pairwise differences in the post hoc analysis (Figure 2A).

#### KLH-IgG

*Overall antibody response* Overall, KLH-IgG titers were significantly affected by housing (F(1.52) = 7.4, *P* < 0.01), sampling day (F(6.307) = 2.7, *P* < 0.05), and their interaction (F(6.307) = 2.3, *P* < 0.05). Post hoc analysis showed that only for enriched pigs titers on day 52 were significantly higher than those on day 46, whereas barren housed pigs showed no day effect on KLH-IgG titers (Figure 1B).

*Housing effect on antibody titers before infection* Before transport, at 38 days of age, KLH binding IgG titers were higher in barren housed pigs than in enriched housed pigs (F(1.54) = 16.5, *P* < 0.001, Figure 1B).

*Housing effect on changes in antibody titers after transport and relocation* Housing affected the change of KLH-IgG titers before and after transport (delta 44–38) (F(1.54) = 5.0, *P* < 0.05) (Table 1). KLH-IgG increased in enriched housed pigs, whereas KLH-IgG decreased in barren housed pigs (Table 1).

*Effects of infection and its interaction with housing on changes in antibody titers after infection* There was no effect of PRRSV nor *A. pleuropneumoniae* infection on KLH-IgG titers (Figures 1B, 2B).

#### IgM and IgG binding MBP

##### MBP-IgM

*Overall antibody response* Generally, MBP-IgM titers were affected by housing (F(1.52) = 3.6, *P* < 0.05), infection (F(1.52) = 4.0, *P* < 0.10), sampling day (F(6.307) = 62.3, *P* < 0.001), and by the housing × sampling day (F(6.307) = 2.2), infection × sampling day (F(6.307) = 4.4), and housing × infection × sampling day (F(6.307) = 2.2) interactions (*P* < 0.05, see Figure 1C).

*Housing effect on antibody titers before infection* At 38 days of age, MBP-IgM titers were affected by housing (F(1.54) = 10.0, *P* < 0.01), with higher titers in barren compared to enriched housed pigs (Figure 1C).

*Housing effect on changes in antibody titers after transport and relocation* After transport, the deltas in MBP-IgM titers between day 44 and 38 were affected by housing (F(1.54) = 5.4, *P* < 0.05), as well. MBP-IgM increased more in enriched housed pigs than in barren housed pigs (Table 1).

*Effects of infection and its interaction with housing on changes in antibody titers after infection* Changes in titers following infection with PRRSV were affected by infection (F(1.52) = 15.1, *P* < 0.001), delta-day (F(2.99) = 55.8, *P* < 0.001), their interaction (F(2.99) = 3.8, *P* < 0.05) and the housing × infection × delta-day interaction (F(2.99) = 4.0, *P* < 0.05). Post hoc analysis showed that the increase in MBP-IgM titers following PRRSV infection (delta from titers on day 44) was higher between delta 52–44 than between delta 46-44, with delta 48–44 in between, except for the enriched infected group where titer changes between day 52 and 44 were also higher than those over delta 48–44. Thus, only in infected enriched pigs MBP-IgM titers continued to increase from day 48 to day 52. Barren and enriched controls did not differ in titer changes, and barren infected pigs did not differ from barren controls for any time trajectory. Enriched infected pigs, however, had a significantly higher increase in titers from day 44 to day 52 after PRRSV than barren infected pigs, and than both barren and enriched controls (Figure 2C).

After infection with *A. pleuropneumoniae*, there was no infection effect on MBP-IgM deltas (delta 53–52, 54–52), but only of the interaction between housing and delta-day (F(1.50) = 9.9, *P* < 0.01), with a lower decrease in titers from delta 54–52 than from delta 53–52 in barren pigs only (Figure 2C).

##### MBP-IgG

*Overall antibody response* Overall, only sampling day (F(6.307) = 19.4) and the interaction between housing and sampling day (F(6.307) = 4.2) affected the levels of MBP binding IgG in the serum (*P* < 0.001). Post hoc analysis showed that in enriched housed pigs titers of MBP-IgG were lower on day 38 than those on each other sampling day, whereas in barren pigs titers on day 38 only differed from those on days 53, and 54 and tended to differ from titers at day 52 (Figure 1D).

*Housing effect on antibody titers before infection* On day 38, MBP-IgG titers were higher in barren pigs than in enriched housed pigs (housing effect, F(1.54) = 12.7, *P* < 0.001, Figure 1D).

*Housing effect on changes in antibody titers after transport and relocation* Housing also affected MBP-IgG changes after transport (delta 44–38) (F(1.54) = 7.9, *P* < 0.01), with MBP-IgG titers increasing more in enriched compared with barren housed pigs (Table 1).

*Effects of infection and its interaction with housing on changes in antibody titers after infection* After infection with PRRSV, there was no housing or infection effect on MBP-IgG, but after infection with *A*. *pleuropneumoniae*, there was a housing effect on MBP-IgG (F(1.50) = 11.6, *P* < 0.01), with higher titers in barren than in enriched housed pigs (Figure 2D).

#### IgM and IgG binding PC-BSA

##### PC-BSA-IgM

*Overall antibody response* Generally, PC-BSA-IgM titers were affected by housing (F(1.52) = 4.2), *P* < 0.05), sampling day (F(6.307) = 124.0, *P* < 0.001), their interaction (F(6.307) = 5.1, *P* < 0.001), infection (F(1.52) = 13.1, *P* < 0.001), and the housing × infection × sampling day interaction (F(6.307) = 2.8, *P* < 0.05) (Figure 1E).

*Housing effect on antibody titers before infection* At 38 days of age, PC-BSA-IgM titers were affected by housing (F(1.54) = 21.8, *P* < 0.001); barren housed pigs had higher levels of PC-BSA-IgM than enriched housed pigs (Figure 1E).

*Housing effect on changes in antibody titers after transport and relocation* Changes in PC-BSA-IgM titers after transport (delta 44–38) were affected by housing (F(1.54) = 11.1, *P* < 0.01) as well; in enriched housed pigs, PC-BSA-IgM titers increased more than in barren housed pigs (Table 1).

*Effects of infection and its interaction with housing on changes in antibody titers after infection* After infection with PRRSV, delta PC-BSA-IgM (delta 46-44, 48–44, and 52–44) tended to be affected by the interaction between infection and housing (F(1.52) = 3.1, *P* < 0.10), by delta-day (F(2.99) = 19.5, *P* < 0.001) and by the housing × infection × delta-day interaction (F(2.99) = 2.9, *P* < 0.10). Post hoc analysis showed that the changes between 52 and 44 were significantly higher than the changes between 46 and 44 for both barren control pigs and enriched infection pigs. There was no infection effect after infection with *A. pleuropneumoniae* (Figure 2E), but an effect of delta-day (F(1.50) = 4.0, *P* < 0.05) and a tendency for a housing × delta-day effect (F(1.50) = 3.6, *P* < 0.10).

##### PC-BSA-IgG

*Overall antibody response* Overall, PC-BSA-IgG titers were affected by sampling day (F(6.307) = 32.4, *P* < 0.001), and their interaction (F(6.307) = 2.2, *P* < 0.05). Unlike the other antibodies, titers of PC-BSA-IgG decreased over time (Figure 1F). Post hoc analysis showed that for both barren and enriched groups, levels of PC-BSA-IgG on day 38 were higher than on other sampling days.

*Housing effect on antibody titers before infection* There was no housing effect on PC-BSA-IgG on day 38.

*Housing effect on changes in antibody titers after transport and relocation* After transport, the delta PC-BSA-IgG values between day 44 and day 38 were affected by housing (F(1.54) = 9.0, *P* < 0.01). PC-BSA-IgG decreased less in enriched housed pigs than in barren housed pigs (Table 1).

*Effects of infection and its interaction with housing on changes in antibody titers after infection* There was no infection effect (Figures 1F, 2F).

#### Relationships between viral clearance and NA(A)b

**Figure 3:**
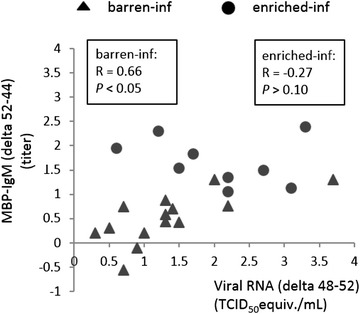
**The correlation between viral clearance and NA(A)b.** The correlation between the changes of MBP-IgM from day 44 to day 52 and the reduction of viral RNA from day 48 to day 52 for barren and enriched pigs infected with PRRSV.

The titers of IgG NA(A)b and IgM NA(A)b except MBP-IgM on day 44 and their increase until day 52 did not correlate with viral RNA levels. Increasing MBP-IgM levels (delta 52–44) were positively correlated with the decrease in viral RNA from day 48 to day 52 (R = 0.53, *P* < 0.05; see Figure 3). However, the latter relationship was stronger in barren (R = 0.66, *P* < 0.05) than in enriched housed pigs (R = −0.27, *P* > 0.10) as the two correlation coefficients significantly differed (*P* < 0.05). No other correlations were found.

## Discussion

In this study, we investigated the effect of environmental enrichment versus barren housing on the level of NA(A)b binding KLH, MBP, and PC-BSA prior to transport and relocation, after transport and after infection with PRRSV and *A. pleuropneumoniae*. In general, all IgM antibody titers increased over time, which may reflect an aging effect [16]. It has been proposed that IgM antibodies binding auto-antigens such as PC are usually present, and enough IgM is available to prevent inflammation, for instance by activating the classical complement pathway [19], and consequently IgG is not formed. In the current study, IgG binding to PC-BSA decreased over time, which is in line with the previous study [13]. Whether this also reflected decreasing maternal IgG antibodies remains unknown. IgM antibodies in pigs are most likely not from maternal origin, thus housing effects and infection effects on IgM most likely reflect effects on the pigs’ immune system. Our study shows that housing before transport affected NA(A)b levels in the blood, with generally significantly higher levels of IgM and IgG binding KLH, MBP and PC-BSA in barren housed pigs. Infection only affected IgM, but not IgG natural (auto-)antibodies, and did so in a housing-dependent manner.

### Housing effects

Housing or its interaction with sampling day affected all NA(A)b levels except KLH-IgM. In line with this, effects of housing on NA(A)b binding KLH (IgG, [16]), MBP and PC-BSA (IgM, [13]) have previously been found. Notably, before infection, barren housed pigs had higher levels of NA(A)b than enriched housed pigs, except for PC-BSA-IgG. This is in contrast with previous studies in which generally lower NA(A)b titers were found for barren housed pigs, with the exception of PC-BSA-IgG [13, 16]. There could be several reasons for this discrepancy. First, in the present experiment, pigs were housed in enriched or barren conditions, starting directly after birth instead of starting after weaning. Therefore pigs in the present study had a longer experience with enriched conditions than the pigs in the previous studies. Second, unlike the previous experiments, enriched housed pigs in the present study were not only provided with ample substrates to root in, but also with extra space, which seems to be an important enrichment factor [25, 26]. Finally, blood samples were taken at a younger age as compared to previous studies.

The changes in antibody titres from day 38 (before transport and relocation) to day 44, just before infection (delta transport effect) were significantly different between barren and enriched housed pigs too. Higher deltas (higher increases and lower decrease) were found in the enriched housed pigs, whereas antibody titers on day 38 and 44 were not much different for barren housed pigs, which already had higher titers on day 38. It can be speculated that the increases in antibody titers found in particularly enriched housed pigs is related to the stress of transport and relocation experienced by these pigs, as we previously found increased anti-PC-BSA antibody titers after regrouping stress [13], but age effects cannot be fully excluded. In our previous study, however, both barren and enriched housed pigs showed such increases following regrouping [13].

### Infection effects

Effects of infection with PRRSV on the titers on day 44 up till day 52 were found for IgM binding MBP and PC-BSA. Lobo et al. [22, 27] also found that IgM binding leukocytes, but not IgG can limit HIV infection in an antigen-nonspecific fashion. The reason could be that the functional activity of IgG is blocked by poly-reactive IgM with anti-idiotypic activity [28]. It has been proposed that natural IgG-producing B cells, unlike natural IgM-producing B cells, are in an inactive state at birth, and these B cells start to produce IgG after exposure to bacterial or foreign antigens [29, 30], and thus it may take a long time before significant levels of IgG can be detected [31]. It is, therefore, not surprising that the effect of IgG on infection was not detected within the short infection period in this study. Remarkably, in the current study, the effects of PRRSV infection on IgM NA(A)b titers were housing-dependent. Only in infected enriched pigs, MBP-IgM titers continued to increase from day 4 to day 8 after infection (day 48–52 of age), and infected pigs from enriched housing showed a higher increase in titers starting directly after PRRSV until 8 days after infection than controls and barren infected pigs. Thus, enriched infected pigs showed a higher and more prolonged increase from infection onwards in MBP-IgM. A previous study on the same pigs revealed that infection characteristics differed for barren and enriched housed pigs. Four days after PRRSV infection (day 48) viral RNA amount was equally increased in both barren infected and enriched infected pigs, whereas 8 days after infection (day 52), enriched infected pigs had significantly lower viral RNA. Moreover, enriched housed pigs were subsequently less likely to develop lung lesions following *A*. *pleuropneumoniae* infection [8].

Remarkably, in barren, but not enriched housed pigs a correlation was found between the increase in MBP-IgM antibody titers from day 44 to 52 and the reduction in viral RNA between day 48 and 52. Thus, barren housed pigs with a relatively higher increase in MBP-IgM following infection, showed a faster reduction of viral RNA in serum. This correlation was not found in enriched pigs, who had both already higher MBP-IgM titers (in this study) and a faster reduction in viral RNA [8]. Whether or not the IgM NA(A)b play a role in defence against the virus, remains to be elucidated.

Pigs were sacrificed shortly after *A*. *pleuropneumoniae* infection on day 52, and no effect of infection of the bacteria was found on the changes in NA(A)b up till day 54, except for KLH-IgM.

The results of the current study are difficult to explain. NA(A)b levels have been proposed to be independent from the environment [19]. However, in the current study, NA(A)b, particularly of the IgM isotype, binding all three antigens were affected by housing conditions, and, moreover infection with PRRSV affected MBP-IgM NA(A)b in a housing-dependent manner. Our data suggested that environmental enrichment may facilitate immune sensitivity in a non-antigen specific fashion resulting in higher NA(A)b levels following viral infection. In poultry, levels of auto-IgM showed a relatively high heritability as opposed to auto-IgG suggesting that IgM is less affected by the environment than IgG [32]. It remains to be elucidated whether the higher or lower antibody levels in the present study with pigs reflect the usage of antibody in response to cell or tissue damage due to stress as a homeostatic response [14], or reflect a non-antigen specific B cell activation [19]. Either way, this study suggests that levels of auto-antibodies including IgM, are affected by the environment (housing) and/or infection, and therefore may provide additional information of the physiological response of pigs to stress in general. Remarkably, the enriched housed pigs were less prone to develop lesions caused by a combination of PRRSV and *A. pleuropneumoniae* [8]. Whether this rests on a higher sensitivity of NA(A)b to the environment or changing NA(A)b levels reflect sensitivity of the individual to the environment remains to be established.

In conclusion, type of housing affected serum levels of NA(A)b binding KLH, MBP and PC-BSA. Infection with PRRSV only affected the IgM, but not the IgG isotype. In this study, barren housed pigs had higher levels of NA(A)b, except for PC-BSA-IgG, than enriched housed pigs before transport and infection. However, NA(A)b levels of enriched housed pigs responded more to transport and relocation stress than barren housed pigs. Besides, enriched and barren housed pigs responded differently to PRRSV infection. NA(A)b may thus be affected by infection, and the effect of infection on NA(A)b is influenced by housing conditions. More research is warranted to investigate the mechanisms by which environmental enrichment affects NA(A)b and the role of NA(A)b in the defence of infection.
